# Leonard’s Physician Pocket Day-Book

**Published:** 1894-01

**Authors:** 


LEONARD'S PHYSICIAN'S POCKET DAY-BOOK. Bound in Red Morocco, with Flap, Pocket and Pencil Loop, Price Postpaid, $1.00. Published Annually by the ILLLUSTRATED MEDICAL JOURNAL CO., Detroit, Mich.

This popular day-book is now in its 16th year of publication. It is good for *thirteen* months from the first of *any* month that it may be begun, and accommodates charges for fifty patients daily for that time, besides having cash department, and complete obsteric records. There is space for the diagnosis of each case, or for brief records of the treatment adopted, following each name space. It has the usual *printed matter*, such as: Dose List; Poisons and Antidotes; Urinary Tests; Exanthematics; Disinfectants; Weights and Measures.

